# Estimating institutional physician turnover attributable to self-reported burnout and associated financial burden: a case study

**DOI:** 10.1186/s12913-018-3663-z

**Published:** 2018-11-27

**Authors:** Maryam S. Hamidi, Bryan Bohman, Christy Sandborg, Rebecca Smith-Coggins, Patty de Vries, Marisa S. Albert, Mary Lou Murphy, Dana Welle, Mickey T. Trockel

**Affiliations:** 1 Department of Psychiatry and Behavioral Sciences, Stanford University, 401 Quarry Road, Office 1320, Stanford, CA 94305 USA; 2 Stanford Medicine Well MD Center, Stanford University, Stanford, CA 94305 USA; 3 Stanford Health Care, 300 Pasteur Dr, Stanford, CA 94305 USA; 4 Stanford Children’s Health | Lucile Packard Children’s Hospital, Pediatric Rheumatology, 730 Welch Rd, Palo Alto, CA 94304 USA; 5 Stanford Emergency Department, 300 Pasteur Dr, Palo Alto, CA 94304 USA; 6 Stanford Children’s Health | Lucile Packard Children’s Hospital, 725 Welch Rd, Palo Alto, CA 94304 USA; 7 The Risk Authority Stanford, 1510 Page Mill Road, Palo Alto, CA 94304 USA

**Keywords:** Burnout, Physician well-being, Turnover, Intent to leave

## Abstract

**Background:**

Awareness of the economic cost of physician attrition due to burnout in academic medical centers may help motivate organizational level efforts to improve physician wellbeing and reduce turnover. Our objectives are: 1) to use a recent longitudinal data as a case example to examine the associations between physician self-reported burnout, intent to leave (ITL) and actual turnover within two years, and 2) to estimate the cost of physician turnover attributable to burnout.

**Methods:**

We used de-identified data from 472 physicians who completed a quality improvement survey conducted in 2013 at two Stanford University affiliated hospitals to assess physician wellness. To maintain the confidentially of survey responders, potentially identifiable demographic variables were not used in this analysis. A third party custodian of the data compiled turnover data in 2015 using medical staff roster. We used logistic regression to adjust for potentially confounding factors.

**Results:**

At baseline, 26% of physicians reported experiencing burnout and 28% reported ITL within the next 2 years. Two years later, 13% of surveyed physicians had actually left. Those who reported ITL were more than three times as likely to have left. Physicians who reported experiencing burnout were more than twice as likely to have left the institution within the two-year period (Relative Risk (RR) = 2.1; 95% CI = 1.3–3.3). After adjusting for surgical specialty, work hour categories, sleep-related impairment, anxiety, and depression in a logistic regression model, physicians who experienced burnout in 2013 had 168% higher odds (Odds Ratio = 2.68, 95% CI: 1.34–5.38) of leaving Stanford by 2015 compared to those who did not experience burnout. The estimated two-year recruitment cost incurred due to departure attributable to burnout was between $15,544,000 and $55,506,000. Risk of ITL attributable to burnout was 3.7 times risk of actual turnover attributable to burnout.

**Conclusions:**

Institutions interested in the economic cost of turnover attributable to burnout can readily calculate this parameter using survey data linked to a subsequent indicator of departure from the institution. ITL data in cross-sectional studies can also be used with an adjustment factor to correct for overestimation of risk of intent to leave attributable to burnout.

## Background

The increasing prevalence of burnout among physicians and its negative effects on health care quality are well documented [1, 2]. Prevention programs implemented at the organizational level are likely to be more effective in reducing physician burnout than programs that only target individual physicians (e.g. time management, stress reduction and resilience building skills) [3]. Accurate information on the economic cost of physician burnout to health care organizations may help motivate systematic organizational level efforts to improve physician wellbeing [4]. Medical errors, malpractice suits, physician turnover, reductions in clinical work hours, and lower patient satisfaction are some the consequences of physician burnout that result in increased organizational costs [4–7]. Many factors that have been identified as contributing factors to burnout- such as low professional satisfaction, medical-legal, health, and financial issues, lack of alignment between personal and organizational values and challenging practice arrangements [8–10] are also factors that contribute to physicians’ turnover or early retirement [1, 11–13].

Although many reports have demonstrated associations between burnout and intent to leave, [7, 14–23] only one study has examined the association between physician burnout and actual subsequent turnover in an academic medical center in the US [6]. In terms of estimated cost of physician burnout, there is only one study that has estimated the cost of departure, based on intent to leave data, attributable to burnout in Canada in 2014 [17].

The current gold standard to assess burnout out is the Maslach Burnout Inventory-Human Services Survey (MBI-HSS), with 22 items measuring three common symptoms of burnout: emotional exhaustion, depersonalization, and lack of personal accomplishment. Another commonly used measure of burnout in physicians is a validated non-proprietary one-item self-reported burnout scale [24–27] which has a strong positive correlation with the emotional exhaustion sub-scale of the MBI [26, 27]. It is the emotional exhaustion subscale of MBI that has been shown to be the strongest predictor of physician attrition [6] and intent to leave the institution [12, 19, 28]. Burnout indicated by the one-item self-reported scale is also associated with physicians’ intent to leave, [15, 23] while no associations have been found between depersonalization and physician attrition [6] or intent to leave [19, 28]. Emotional exhaustion correlates with anxiety and depression in physicians [29, 30]. Previous reports examining the effect of physician burnout on intent to leave and/or actual departure have not adjusted for the effects of anxiety and depression.

The primary objective of our study is to examine the associations of physician self-reported burnout with intent to leave and actual turnover, using a recent longitudinal data case example, both before and after adjusting for anxiety and depression. The second objective is to estimate the cost of physician turnover attributable to burnout. The third objective is to explore the relationship between intent to leave and actual turnover, and thus to assess the feasibility of using intent to leave data as a proxy for turnover data when data on actual turnover is not available.

## Methods

### Study design

Between April–May 2013, the Stanford Committee for Professional Satisfaction and Support (SCPSS) conducted a survey to assess wellness of physicians affiliated with Stanford University. The purpose of the 2013 Physician Wellness Survey was to help SCPSS develop, implement, and evaluate quality improvement projects and interventions to support physicians’ professional fulfillment at Stanford and to prevent burnout [31]. We used de-identified data from this survey for this study. This analysis of de-identified data was deemed to be exempt (a non-human subject research study) by Stanford University’s Institutional Review Board (IRB). Because we used a de-identified database, demographic data such as race, age, gender, clinical specialty (except for surgical vs. non-surgical specialty) and academic rank were not available and therefore not included in our analysis.

### Setting and participants

**Fig. 1:**
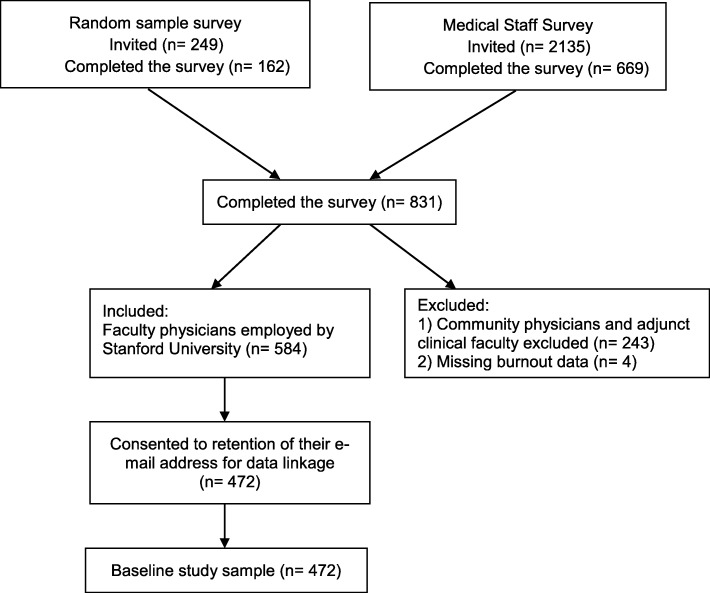
Study flow Diagram

Approximately 10 % (*n* = 249) of physicians credentialed at one or both of two Stanford affiliated hospitals were randomly selected from the medical staff and offered a $25 gift certificate to complete the Physician Wellness Survey, of which 162 (65% response rate) did so. In addition to the random sample, 669 (31% response rate) of the remaining 2135 medical staff members from both hospitals completed the subsequent survey of all physicians. All medical staff - including both employed and non-employed (adjunct) faculty (2135) as well as community physicians (249) - were invited to participate in this survey (2135 + 249 = 2384). The overall response rate for this survey was 34.9% ([162 + 669]/2384). Scores on survey measures from the two samples did not differ significantly. For this analysis, we included faculty physicians employed by Stanford University from both samples who consented to retention of their e-mail address, which enabled us to link their survey responses across time and to other data (Fig. 1, *n* = 472).

The Physician Wellness Survey included previously developed and validated measures to assess self-reported burnout [25–27] and intent to leave [23, 32, 33]. We used the NIH Patient-Reported Outcomes Measurement Information System (PROMIS®) Short Forms to assess sleep-related impairment [34], depression, and anxiety [35, 36]. Sleep-related impairment scores range from a minimum of 8 to maximum of 40, and anxiety and depression scores each range from 4 to 20. The NIH PROMIS guidelines can be used to identify T-scores that correspond to raw scores of each scale. T-scores of 50 or higher (with a standard deviation of 10) in these instruments are more likely to represent people with poorer health than the general population [37–39]. Turnover data in 2015 was compiled by a third party custodian of the data using the medical staff directory. The cost of recruitment and start-up costs for new faculty hires was provided by the Stanford School of Medicine Chief Financial Officer.

### Statistical methods

We analyzed data using IBM SPSS Statistics 23 (IBM Corp, Armonk, NY, USA). All reported *p*-values are 2-tailed, with statistical significance set at *p* <  0.05. We calculated relative risk (RR) ratio for unadjusted relationships between burnout and turnover, between burnout and intent to leave, and between intent to leave and turnover. We used logistic regression to estimate odds ratio (OR) effects adjusted for potentially confounding variables including hours worked per week category, [1, 40, 41] surgical specialty (yes, no), [20, 42] and sleep-related impairment, anxiety and depression scales (continuous variables) [43].

## Results

Our baseline study sample includes 472 physicians (Fig. 1). Sixty-one (13%) physicians in the baseline sample in 2013 had left Stanford by 2015. As baseline 26% of physician reported experiencing burnout and 133 (28%) reported intent to leave within the next 2 years (Table 1). The majority of physicians in our sample reported working over 50 h per week. Based on the PROMIS guidelines, the closest T-scores and standard errors corresponding to the raw score of sleep-related impairment falls between 50.3 ± 2.7 and 51.6 ± 2.6, [37] for anxiety falls between 48.0 ± 3.6 and 51.2 ± 3.1, [38] and for depression falls between 49.0 ± 3.2 and 51.8 ± 2.7 [37, 39].

**Table 1:** Characteristics of physicians in the baseline study sample, and associations between physician attrition and burnout

	Total (n = 472)	Did not leave (*n* = 411)			Left (*n* = 61)	
	n (%)	n (%)			n (%)	
Intent to leave within 2 years Missing	133 (28) 6 (1)	100 (24) 4 (1)			33 (54) 2 (3)	
Hours worked per week categories						
Very high work hours (≥ 64 h)	127 (27)	109 (27)			18 (30)	
High work hours (51–63 h)	143 (30)	128 (31)			15 (25)	
Moderate work hours (41–50 h)	111 (24)	98 (24)			14 (23)	
Low works hours (≤40 h)	85 (18)	72 (18)			13 (21)	
Missing	6 (1)	5 (1)			1 (2)	
Surgical specialty	79 (17)	65 (16)			14 (23)	
Burned out	123 (26)	97 (24)			26 (43)	
	n	Mean (SD)	n	Mean (SD)	n	Mean (SD)
Anxiety	467	5.8 (2.6)	407	5.7 (2.5)	60	6.2 (3.0)
Depression	464	5.5 (2.5)	404	5.4 (2.4)	60	6.0 (3.1)
Sleep-related impairment	472	16.8 (6.6)	411	16.7 (6.5)	61	18.0 (7.3)

*Abbreviations*: *n* number, *SD* Standard Deviation

### Burnout and turnover

The associations between burnout and turnover are reported in Table 2. After adjusting for surgical specialty, work hour categories, sleep-related impairment, anxiety, and depression, physicians who experienced burnout in 2013 had 168% higher odds (Odds Ratio (OR) = 2.68, 95% CI: 1.34–5.38, Table 2) of leaving Stanford by 2015 compared to those who did not experience burnout. Surgical specialty, work hours category, sleep-related impairment, anxiety and depression were not statistically significant predictors of physician turnover.

**Table 2:** Association between physician turnover and burnout

	OR (95% CI)	*p*-value
Unadjusted Model	2.40 (1.38–4.19)	0.002
Adjusted Model 1^a^	2.41 (1.38–4.21)	0.002
Adjusted Model 2^b^	2.82 (1.56–5.10)	0.001
Adjusted Model 3^c^	2.77 (1.47–5.25)	0.002
Adjusted Model 4^d^	2.68 (1.34–5.38)	0.005

Abbreviations: *OR* Odds Ratio, *CI* Confidence Interval

^a^ Adjusted for Surgical Specialty

^b^ Adjusted for Surgical Specialty, Hours Worked per Week Category

^c^ Adjusted for Surgical Specialty, Hours Worked per Week Category, and Sleep-Related Impairment

^d^ Adjusted for Surgical Specialty, Hours Worked per Week Category, Sleep-Related Impairment, Anxiety, and Depression

### Estimating the economic cost of burnout

**Fig. 2:**
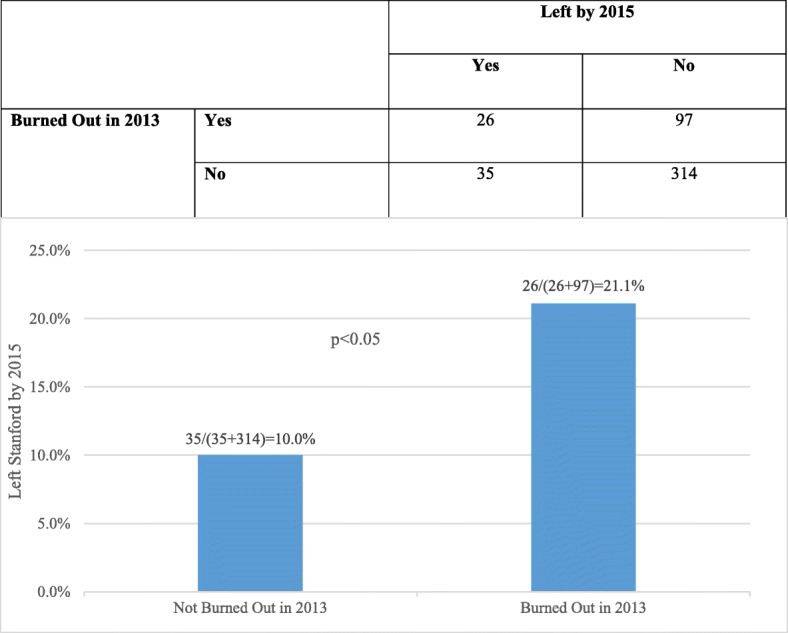
Physician attrition and burnout. Attributable risk of burnout = 21–10% = 11%. Attrition of 11% of those who were burned out may be attributed to burnout, while attrition of 10% may be attributed to other causes, such as a compelling career opportunity elsewhere, or planned retirement

Twenty one percent of physicians who reported one or more symptoms of burnout in 2013 had left by 2015 compared with only 10% of those without burnout symptoms in 2013 (Fig. 2, RR = 2.1; 95% C: 1.3–3.3). Therefore, departure of 11% of those who were burned out 2 years earlier may be attributed to burnout [attributable risk = 21–10% = 11%]. The overall rate of burnout in 2013 was 26%. If these results generalize to all physicians currently employed by the Stanford School of Medicine (*n* = 2023 out of 2678 total medical staff − 655 are community physicians and/or adjunct clinical faculty members not employed by the School of Medicine), then over the next 2 years the departure of 58 faculty physicians will be attributable to burnout [0.11*0.26*2023 = 58]. The cost of physician recruitment and start-up (not including housing) at this AMC ranges between $268,000 - $957,000 per physician based on specialty, experience and expertise. Therefore, the minimum estimated two-year economic loss due to physician departure attributable to burnout will range between $15,544,000 (58* $268,000) and $55,506,000 (58* $957,000).

### Intent to leave versus actual turnover

In 2013, 133 (28%) of physicians indicated intent to leave their current practice within 2 years. Two years later, in 2015, 33 physicians out of 133 with intent to leave (24.8%) had left Stanford compared to 26 physicians who left out of 333 without intent to leave (7.8%) (Relative Risk (RR) = 3.2, 95% CI: 2.0–5.1). Overall, 61 of the 472 physicians (13%) who completed the 2013 survey had left Stanford by 2015.

### Burnout and intent to leave

In the fully adjusted model (Table 3), those who were experiencing burnout had 4.86 times higher odds of intent to leave compare to those who were not experiencing burnout. We also found that for every one point (range: 4–20) increase in depression score, physicians had 22% greater odds of intent to leave (Table 4). There were no statically significant associations between intent to leave and surgical specialty, work hour category, sleep-related impairment or anxiety scores (Table 4).

**Table 3:** Association between physicians’ intent to leave and burnout

	OR (95% CI)	*p*-Value
Unadjusted Model	6.48 (4.11–10.21)	*< 0.001*
Adjusted Model 1^a^	6.48 (4.11–10.20)	*< 0.001*
Adjusted Model 2^b^	7.06 (4.38–1.40)	*< 0.001*
Adjusted Model 3^c^	6.63 (3.98–11.03)	*< 0.001*
Adjusted Model 4^d^	4.86 (2.81–8.39)	*< 0.001*

*Abbreviations*: *OR* Odds Ratio, *CI* Confidence Interval

^a^ Adjusted for Surgical Specialty

^b^ Adjusted for Surgical Specialty, Hours Worked per Week Category

^c^ Adjusted for Surgical Specialty, Hours Worked per Week Category, and Sleep-Related Impairment

^d^ Adjusted for Surgical Specialty, Hours Worked per Week Category, Sleep-Related Impairment, Anxiety, and Depression

**Table 4:** Association between intent to leave and burnout - details of Model 4

	OR	(95% CI)	*p*-Value
Burned Out	4.86	2.81–8.39	*< 0.001*
Surgical Specialty	1.39	0.75–2.57	0.29
Very High Work Hours	0.51	0.25–1.05	0.07
High Work Hours	0.60	0.30–1.20	0.15
Moderate Work Hours	0.75	0.37–1.51	0.42
Low Work Hours (reference group)	1	–	–
Sleep-Related Impairment	0.99	0.95–1.03	0.63
Anxiety	1.01	0.89–1.16	0.85
Depression	1.22	1.06–1.39	*0.004*

*Abbreviations*: *OR* Odds Ratio, *CI* Confidence Interval

Among physicians who reported one or more symptoms of burnout in 2013, 58.7% indicated intent to leave their practice within 2 years, compared with only 18% of those without burnout symptoms (RR = 3.3; 95% C:2.5–4.3). Therefore, intent to leave of 41% of those who were burned out may be attributed to burnout [attributable risk = 58.7–18% = 40.7%] which is about 3.7 times higher than the actual observed rate of subsequent turnover attributable to burnout.

## Discussion

The results of our analysis indicate that 1) physicians who are experiencing burnout are more than twice likely to leave their practice, and that the effect of burnout on turnover is independent of personal factors such as anxiety or depression; 2) the economic cost of turnover attributable to physician burnout is high, and; 3) intent to leave is a strong predictor of actual departure. Those who reported intent to leave the institution were more than three times as likely to leave within 2 years, compared to those who did not report intent to leave.

In this study sample, physicians had modestly higher scores for sleep-related impairment, and similar to median scores for anxiety, and depression to median scores from PROMIS reference populations (the 2000 General US Census and a clinical sample for sleep-related impairment, and the 2000 General US Census for anxiety and depression) [37–39]. Although depression was a significant predictor of intent to leave even after adjusting for other variables, only burnout (an indicator of work-specific distress), rather than depression and anxiety (indicators of general distress) was a significant predictor of actual physician turnover. Similar to other studies, [40, 44] our results suggest that intent to leave is a strong predictor of physician turnover. In our baseline sample, 28% reported intent to leave the institution within 2 years, and after 2 years 13% of physicians had left Stanford. Those who reported intent to leave in 2013 were more than three times as likely to have left by 2015. Intent to leave may be a valid proxy indicator of turnover to calculate organization-specific financial burden of physician turnover attributable to burnout, when data on actual turnover is not available. However, intent to leave attributable to burnout is much higher than actual subsequent turnover attributable to burnout. An adjustment factor for this difference will improve accuracy when estimating financial burden from intent to leave data where turnover data is not yet available. The adjustment factor indicated by the present study is to divide intent to leave attributable to burnout by 3.7 to estimate actual subsequent turnover attributable to burnout. Additional data from a heterogeneous set of health care organizations will help clarify the variance in difference between intent to leave versus actual subsequent turnover attributable to burnout.

Future investigation from a heterogeneous set of health care organizations will also help clarify the variance in economic cost of physician turnover attributable to burnout. Physicians working in academic medical centers often have unique challenges (e.g. academic productivity expectations) and benefits (e.g. opportunities to teach and conduct research) that may affect the relationship between burnout and turnover. The only other published data—to our knowledge—on the association between burnout and turnover indicates an effect of burnout on physician turnover similar in magnitude [6] to the present study finding, which provides some across-organization validity. However, this collaborating data is also from an AMC, whereas most health care organizations in the United States are not AMCs. It is not clear that these findings will generalize accurately in attempts to estimate cost of turnover attributable to burnout in non-AMC organizations. The only published article to date [4] that attempts to use turnover attributable to burnout to build an economic argument for investing in physician well-being relies on unpublished data from the present study [45–47].

The present study is limited to observation of the relationship between burnout and subsequent turnover, which provides necessary but far from sufficient evidence of a causal relationship. In addition to an AMC focus, the present study is also limited to secondary data analysis of completely de-identified evaluation data, without demographic data that might be helpful in assessing generalizability of results. With these limitations in mind, the findings of the present study, coupled with previous evidence of effective strategies to mitigate burnout, [48] suggest feasibility of longitudinal study to determine the effect of interventions to mitigate burnout on cost of physician turnover.

Average turnover rates observed at Stanford (13% in 2 years) are similar to national averages. The Cejka Search and American Medical Group Association Physician Retention Survey results show that the annual rate of physician turnover was about 6% in 2010 and 2011 [49]. If interventions can reduce burnout to half of the current rate, Stanford Medicine may be able to save $7,772,000 to $27,753,000 in physician recruitment costs alone during the subsequent 2 years. These figures based on recruitment cost at Stanford, which are comparable to national averages reported elsewhere [4, 50].

Our estimate of the high cost of turnover attributable to burnout does not include either the loss of revenue due to physician faculty position vacancy or the diminished economic productivity associated with burnout among those who remain employed—such as a reduction in work-hours [51]. Nor does this estimate include a broader range of costs associated with burnout, such as the disruption in the quality and continuity of direct patient care or the reduced health of physicians [9, 52]. Investment in interventions to reduce burnout may pay dividends in all of these domains.

## Conclusion

Our results suggest that the economic cost of physician faculty attrition attributable to burnout is high. This finding may help motivate further investigation to determine the effect of strategies to mitigate burnout on physician turnover and associated economic burden. Academic medical centers offer physicians rewarding careers, including opportunities to develop new frontiers in patient care, create and advance new knowledge, and to educate and mentor trainees. In turn, these institutions benefit immensely from talented physicians who drive high standards in patient care, research, and education. Investing in strategies that prevent or reduce burnout may render economic benefits associated with reduced physician attrition. Perhaps more compelling for AMC-employed physicians and the patients they serve are the benefits that reduced burnout may render to physicians’ personal and professional wellbeing and to the quality of clinical care.
